# Microporous Polyamine (PIM-EA-TB) Modified with Hydrated NiMoO_4_ Enhances the Photocatalytic Reduction of Nitrogen to Ammonia

**DOI:** 10.1021/acsaenm.6c00493

**Published:** 2026-06-05

**Authors:** Lara K. Ribeiro, Ana Beatriz Cardile, Laura O. Libero, Mariolino Carta, Neil B. McKeown, Lucia H. Mascaro, Frank Marken

**Affiliations:** † Department of Chemistry, 201361 Federal University of São Carlos, São Carlos, SP 13565-905, Brazil; ‡ Department of Chemistry, University of Bath Claverton Down, Bath BA2 7AY, U.K.; § Instituto de Síntesis Química y Catálisis Homogénea, 16379 CSIC-Universidad de Zaragoza, C/Pedro Cerbuna 12, Facultad de Ciencias, Zaragoza 50009, Spain; ∥ EaStCHEM, School of Chemistry, 3124 University of Edinburgh, Joseph Black Building, David, Brewster Road, Edinburgh EH9 3JF, Scotland, U.K.

**Keywords:** photoreduction, ammonia, surface engineering, energy conversion, pH neutral catalysis

## Abstract

The earth-abundant photocatalyst NiMoO_4_, readily produced by hydrothermal microwave synthesis, was tested for the light-driven production of ammonia in N_2_-purched water. In blue LED light (λ = 385 nm; 6 mW cm^–2^) and under pH-neutral conditions, ammonia is formed as detected via the indophenol blue method and confirmed by ^15^N isotope experiments. Thermal treatment lowers hydration and affects defect populations in NiMoO_4_ (detected by photoluminescence) as well as suppressing catalysis. Adding methanol as a sacrificial hole quencher increases the ammonia production rate. In exploratory experiments, a surface coating of the NiMoO_4_ photocatalyst with a molecularly rigid microporous polyamine (PIM-EA-TB with p*K*_A_ ∼ 4) is shown to more than double the ammonia production rate. This is tentatively assigned here to a localized pH storage effect, allowing protons from photoanodic processes to be captured by the Tröger base in the polymer backbone and reused in the photocathodic ammonia production.

## Graphical abstract

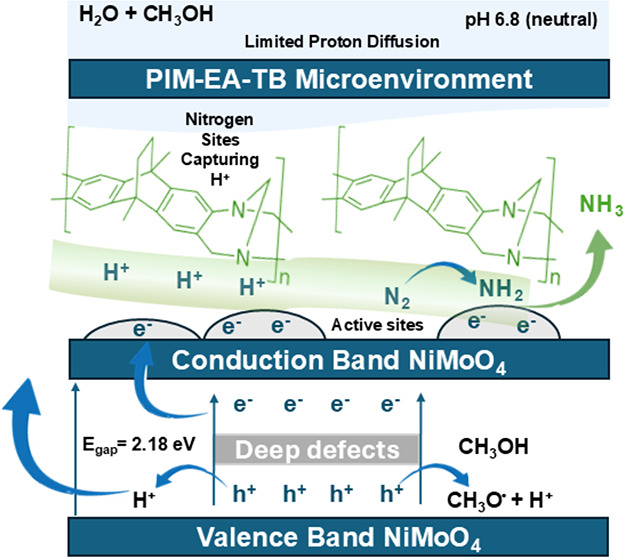

## Introduction

Ammonia is one of the most important requirements for agricultural production and therefore plays a central role in ensuring global food security.^1–3^ The high demand is intrinsically linked to the natural nitrogen cycle, which sustains plant growth and agricultural productivity.^1^ However, population growth and the intensification of food demand at scales exceeding the regenerative capacity of this natural cycle have rendered the supply of bioavailable nitrogen insufficient to meet modern agricultural needs.

In this context, the development of the Haber–Bosch process represented a major technological milestone, enabling the large-scale synthesis of ammonia from its elemental precursors, hydrogen and nitrogen. Despite its historical and industrial relevance, this process presents significant environmental drawbacks, as the hydrogen used is predominantly derived from fossil-based sources, leading to substantial carbon dioxide emissions and energy consumption. Although the Haber–Bosch process was, in its early stages, an exceptionally effective solution to support the expansion of agriculture, the current global scenario, particularly in 2026, is characterized by increasing climate pressure, supply chain pressure, and an urgent need to reduce the carbon footprint of industrial processes. This reality has intensified the search for alternative ammonia synthesis routes that are more sustainable and energetically efficient. In this regard, the catalytic reduction of N_2_ to ammonia has emerged as a promising alternative, although it remains far from achieving efficiencies and scalability compatible with global demands. Among the proposed approaches, photocatalysis has attracted considerable attention due to its ability to directly harness solar energy to drive chemical transformations under mild conditions. By generating photogenerated charge carriers capable of activating chemically inert N_2_ molecules, photocatalytic systems offer the prospect of ammonia synthesis at ambient temperature and pressure, potentially bypassing the high energy input and carbon emissions associated with conventional thermochemical routes.^4–6^

The development of photocatalysts capable of activating and converting N_2_ under mild conditions has become a central topic in contemporary research, particularly when based on strategies that exploit structural engineering,^7^ interfacial effects, and the control of both the surface and subsurface environments of catalytic materials. Molybdates have been implicated in biological nitrogen fixation, and various salts such as iron molybdate,^8^ bismuth molybdates,^9^ W-doped,^10^ and Co-doped^11^ have been shown to act as photocatalysts for ammonia production from nitrogen. Bismuth molybdate has been reported to even produce photoammonia from ambient air.^12^ Among these materials, NiMoO_4_ is particularly attractive due to its suitable band structure, visible-light response, and favorable charge transport properties, which make it a promising semiconductor for photocatalytic reduction reactions.

Nickel molybdate is a known photocatalyst^13^ and has been used previously in photodecolorization,^14–16^ in environmental remediation,^17^ a layered form of Na_2_NiMoO_4_ has also been reported to work in photodegradation of dyes,^18^ NiMoO_4_ has been used in conjunction with graphitic carbon nitride for phenol degradation,^19^ and in conjunction with CoS^20^ or NiS^21^ for photohydrogen evolution. These materials have been extensively investigated with respect to the role of the internal surface or “subsurface” in the dynamics of photogenerated charge carriers, employing strategies such as doping and other approaches developed to optimize their photocatalytic properties.

In parallel, surface coatings have been widely employed to investigate and enhance ammonia production via N_2_ reduction, as the reduction reactions are initiated by the adsorption of N_2_ on the catalyst surface. In this context, TiO_2_@PTFE systems have demonstrated remarkable performance, exhibiting 10.9-fold enhancements compared to the same process with pristine TiO_2_. The critical role of interfacial engineering in promoting N_2_ reduction has been emphasized.^22^ A novel system based on a defective tungsten oxide coupled with an acidic ionic liquid and coated with a carbon shell (W@C) shows approximately 9-fold and 2-fold improvements in ammonia production compared to bare tungsten oxide and the W@C sample, respectively, further emphasizing the effectiveness of coating-assisted interfacial reactivity modulation.^23^ Recently, polymer-based microporous coatings have been suggested to be effective in promoting electrochemical nitrogen reduction.^24^ For instance, MoS_2_ modified with the intrinsically microporous polymer PIM-1 in the form of nanoparticles exhibited an enhanced NH_3_ yield rate of 61.2 μg h^–1^ mg^–1^, which represented a nearly 2-fold improvement compared to pristine MoS_2_. These results underscore the potential of microporous polymers to facilitate N_2_ adsorption and activation at the catalyst interface.

Polymers of intrinsic microporosity (PIMs) were originally developed for gas adsorption and separation applications;^25^ however, owing to their characteristic pore sizes of approximately 0.5–1 nm and their good processability, PIMs have found use across a much broader range of applications.^26,27^ The prototypical PIMs include PIM-1 and PIM-EA-TB. These molecularly rigid polymers can be applied as thin layers on catalyst surfaces, where they modulate (without blocking) the microenvironment near the active sites and increase the local concentration of adsorbates available for reaction. PIM-EA-TB exhibits a potential for mild acid–base interactions (p*K*_A_ ∼ 4), rendering it a functional rather than a passive microporous coating in the catalytic system.^26,28^ The protonation of the tertiary nitrogen would allow capture of protons from photoanodic processes to then locally enhance the coupled photocathodic process.

In this report, the photochemical conversion of nitrogen to ammonia is investigated in water under LED light (λ = 385 nm) with two types of NiMoO_4_. Process enhancements caused by the methanol quencher and by surface modification with a polymer of intrinsic microporosity (PIM-EA-TB) are demonstrated and discussed.

## Experimental Section

### Chemicals

Ni­(NO_3_)_2_·6H_2_O (99%, Aldrich), Na_2_MoO_4_·2H_2_O (98%, Aldrich), sodium perchlorate (≥98%, Aldrich), *para*-(dimethylamino) benzaldehyde (p–C_9_H_11_NO), phenol (≥99%, Aldrich), sodium nitroprusside (≥99%, Aldrich), sodium hydroxide (≥97%, Aldrich), sodium hypochlorite aqueous solution (6–14% active chlorine), concentrated hydrochloric acid (HCl, 37 wt %), phosphoric acid (H_3_PO_4_, 98%), nitric acid (HNO_3_, 65%), chloroform (≥99.8%, Aldrich), and methanol (≥99.6%, Aldrich) were obtained commercially and used without further purification. Nitrogen was purchased from BOC, UK (Pureshield). Ultrapure water (18.2 MΩ cm at 20 °C) obtained from a Thermo Scientific water purification system was used to prepare solutions. The PIM-EA-TB polymer powder (Sigma-Aldrich 918784; monomer weight for C_21_H_20_N_2_ 300 g mol^–1^) was synthesized according to previously reported procedures.^27^ The polymer material has a molecular weight of approximately 70 kDa, a dry density of about 1.1–1.3 g cm^–3^ (equivalent to 3.7–4.3 mmol cm^–3^), and a pore volume ranging from 26% to 30% (fractional free volume, FFV), resulting in an estimated wet density of 1.4–1.7 g cm^–3^.^26,27,29^

### Synthesis

The samples were synthesized using the coprecipitation method, followed by microwave-assisted hydrothermal treatment. For NiMoO_4_ synthesis, 1 × 10^–3^ mol of Na_2_MoO_4_·2H_2_O (98%, Sigma-Aldrich) and 1 × 10^–3^ mol of Ni­(NO_3_)_2_·6H_2_O (99%, Aldrich) were each dissolved in two beakers with 50.0 mL of distilled water. The Ni­(NO_3_)_2_·6H_2_O solution was then slowly added to the Na_2_MoO_4_·2H_2_O solution under constant stirring (∼400 rpm), resulting in the formation of a green precipitate. The mixture was stirred magnetically (∼400 rpm) at room temperature for 10 min. Following this, a microwave-assisted hydrothermal treatment was performed. A Panasonic microwave oven (model NN-ST24QWRUK, 2.45 GHz, 800 W) was modified to operate as a hydrothermal synthesis reactor. The system was adapted to enable ramped temperature control, allowing a gradual and regulated increase in internal temperature. The autoclave was equipped with an analogue pressure gauge (barometer) to monitor internal pressure throughout the reaction. The treatment was conducted at 100 °C for 32 min. After the microwave-assisted hydrothermal step, the resulting powder was then placed in a conventional oven at 300 °C for 2 h in ambient atmosphere, with a heating rate of 10 °C min^–1^. The samples were labeled as NiMoO_4_-100 °C and NiMoO_4_-300 °C based on their specific thermal treatment conditions.

### Materials Characterization

The samples were characterized by XRD on a Rigaku X-ray Diffractometer, model DMax2500PC. The equipment was operated at 40 kV and 60 mA using Cu–Kα radiation (λ = 1.5406 Å). A sweep rate of 0.02°/min was used in the range between 10° and 100°. The powder diffractograms were compared with the diffraction patterns according to the crystallographic sheets of the Inorganic Crystal Structure Database (ICSD). Raman spectra were recorded with an iHR550 spectrometer (Horiba Jobin-Yvon, Kyoto, Japan) coupled to a silicon charged-coupled-device (CCD) detector and an argon ion laser (Melles Griot, Rochester, NY, USA). For this study, a 532 nm laser was employed as the excitation source, and the spectra were collected in the range of 100–1200 cm^–1^. All spectra were collected under ambient conditions with multiple accumulations to improve the signal-to-noise ratio. TA Instruments equipment, using an SDT Q600 cell. PL measurements were performed at room temperature by using a 355 nm laser (Cobolt/Zouk) as the excitation source, focused on a 200 mm spot at a constant power of 5 mW. The morphologies of the samples were observed with an SEM operating at 5 kV (Supra 35-VP, Carl Zeiss, Jena, Germany). UV–vis diffuse reflectance measurements were obtained using a Varian Cary spectrometer model 5G in the diffuse reflectance mode, with a wavelength range of 200–800 nm and a scan speed of 600 nm min^–1^.

### NiMoO_4_ Coating with PIM-EA-TB

The coating with PIM-EA-TB was performed by dispersing 1 mg (or in some cases 2 mg or 5 mg) of the polymer in 2 mL of chloroform under ultrasonic agitation for 15 min. Subsequently, 50 mg of the catalyst (NiMoO_4_-100 °C or NiMoO_4_-300 °C) was added to the dispersion. The mixture was then dried to completely remove the chloroform solvent. Once dried, the modified catalyst material (in powder form) was used for photocatalytic ammonia production tests. For experiments involving different amounts of PIM-EA-TB, the same procedure was followed, adjusting the polymer mass while maintaining the same dispersion protocol.

### N_2_ Reduction Photoreaction

Photocatalytic measurements were performed in a custom-built cell equipped with a quartz window, which allows efficient light penetration into the reaction medium. The N_2_ reduction reaction was carried out under visible-light irradiation using a 385 nm LED (λ = 385 nm, Thorlabs, M385LP1) as the excitation source for photocatalysis with power supply DC2200 and the light intensity was approximately 6 mW cm^–2^. The reaction medium consisted of 100 mL of H_2_O, under continuous magnetic stirring (∼400 rpm) to ensure homogeneous mixing. The temperature was maintained at room temperature (25 °C) throughout the experiment. High-purity N_2_ gas (BOC UK, Pureshield; 99.998% purity) was bubbled into the system at a flow rate of 30 mL min^–1^ for 45 min prior to the reaction to ensure complete saturation of the solution. This gas flow was maintained continuously throughout the 2 h experiment. Acidic trapping solution (5 mmol L^–1^ H_3_PO_4_) was placed in wash bottles downstream of the cell to capture any ammonia released as a gas during the reaction. All photocatalytic reduction experiments, including the dark-control and light-control conditions, were carried out under identical experimental parameters. In Figure 1A,C, a schematic of the N_2_ photoreduction process is provided, along with details of the analytical procedures and the calibration curve used for quantifying the produced ammonia.

**Figure 1:**
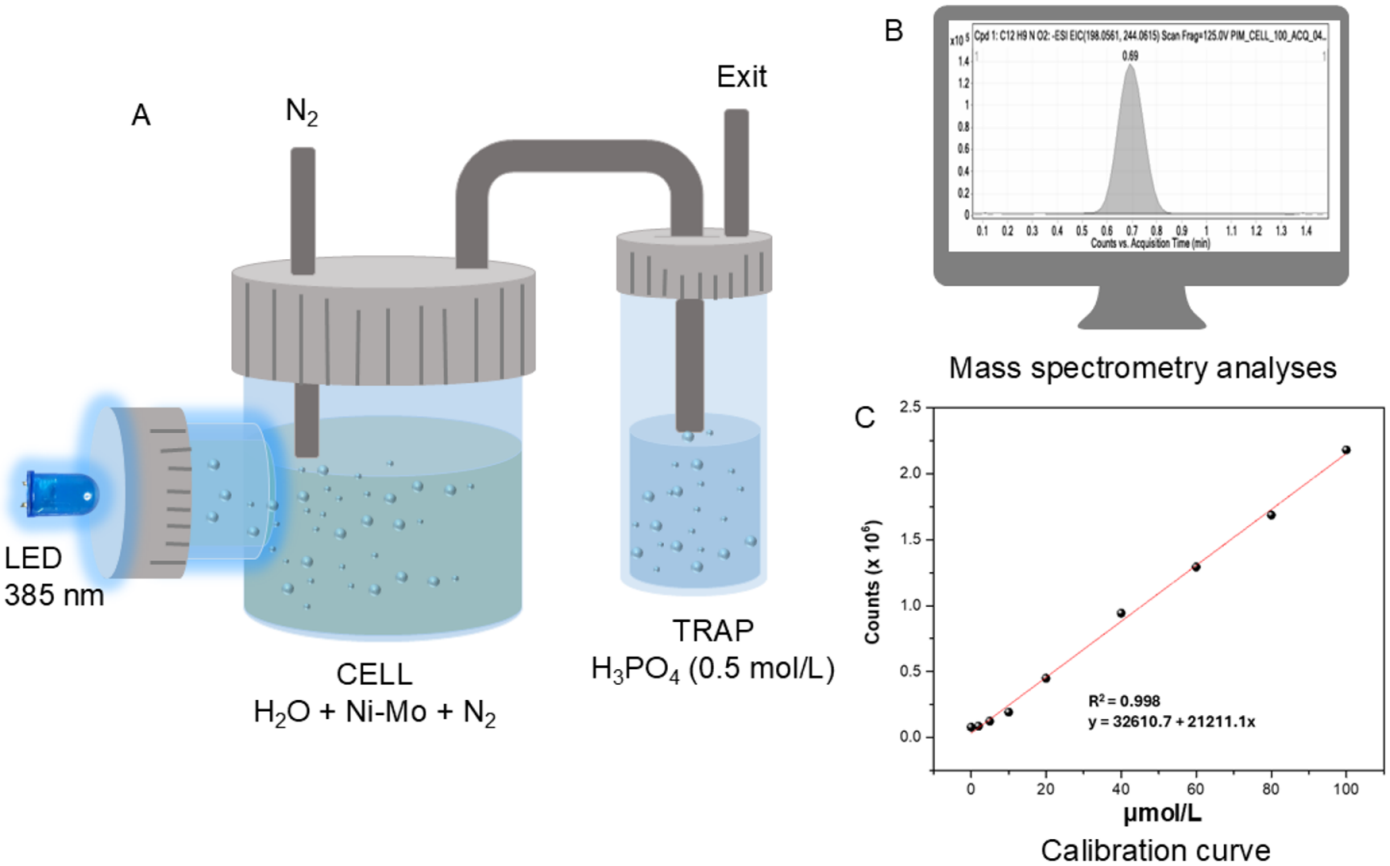
(A) Schematic of the reaction cell equipped with a quartz window for irradiation, N_2_ gas inlet, and outlet to the NH_4_^+^ trap in acidic solution (H_3_PO_4_ 0.5 mol/L). (B) Mass spectrum for the determination of the indophenol blue complex and the corresponding (C) calibration curve used for ammonia quantification.

### NH_3_ Detection

The ammonia yield [NH_3_] in the solution was measured both optically and by using an LC-MS system (Figure 1B). Before the LC-MS analysis, the solution containing NH_3_ was treated following the indophenol blue method. Briefly, two reagent solutions were prepared: Solution 1 consisted of 100 mmol L^–1^ phenol (99%, Aldrich) and 50 mg L^–1^ sodium nitroprusside dihydrate (99.0%, Aldrich) dissolved in ultrapure water. Solution 2 was composed of 0.38 mol L^–1^ dibasic sodium phosphate (99.0%, Aldrich), 0.125 mol L^–1^ sodium hydroxide (97.0%, Aldrich), and 1% (v/v) sodium hypochlorite (6–14% active chlorine). These solutions were used for the colorimetric determination of ammonia via the indophenol blue method. Specifically, aliquots of the reaction solution (200 μL) were mixed with Solution 1 (1 mL) and Solution 2 (1 mL) to promote the formation of indophenol blue. After the color development, the resulting solution was analyzed. For LC-MS measurements, the sample containing the formed indophenol blue derivative was used for injection. A calibration curve for NH_3_ was constructed using the procedure described above in triplicate, on standard NH_4_Cl (99.0%, Aldrich) solutions prepared with NH_4_^+^ concentrations ranging from 0 to 100 μM. The calibration curve for NH_3_ is shown in Figure 1C.

### Methanol as Sacrificial Agent

To investigate the effect of methanol (≥99.6%, Aldrich) concentration (0 mol, 0.01 mol/L, 0.1, and 0.5 mol/L) as a hole scavenger, photocatalytic tests were conducted using different amounts of methanol added to the reaction system. The study was carried out in two stages. In the first stage, methanol was added in varying amounts to evaluate the catalytic activity of the materials synthesized at 100 °C and 300 °C (NiMO_4_-100 and NiMO_4_-300), allowing direct comparison of the two thermal treatments. In the second stage, the best amount of methanol for NH_3_ production was applied to the NiMoO_4_ catalysts previously coated with PIM-EA-TB, aiming to assess the influence of the polymer-functionalized surface on the photocatalytic response under different sacrificial conditions.

### Application of ^15^N Isotope in Experimental Setup

To confirm that the ammonia detected originated from the electrochemical reduction of dinitrogen rather than from external contamination, isotopically labeled ^15^N_2_ (98% atom ^15^N, Cambridge Isotope Laboratories Inc.) was employed as the feed gas. Prior to photocatalysis, the solvent was purged with ^15^N_2_ for 40 min to ensure complete saturation and removal of dissolved air. Control experiments were also performed using ^14^N_2_ under identical conditions to rule out background contributions. After the photochemical reaction, the solvent containing the produced ammonium was collected for analysis. The samples were treated with the indophenol method to convert NH_4_^+^ into a stable indophenol derivative, which was subsequently analyzed by mass spectrometry. The characteristic mass-to-charge ratios of *m*/*z* = 198 (^14^N-indophenolate) and *m*/*z* = 199 (^15^N-indophenolate) were monitored to distinguish between ammonia formed from natural nitrogen and that derived from isotopically labeled nitrogen. This isotopic labeling approach ensured an unambiguous verification of the nitrogen source, providing reliable evidence that the ammonia detected was indeed produced by N_2_ reduction.

## Results and Discussion

### Subsurface Environment Contributions to Catalytic Activity

In heterogeneous catalysis, not only the surface but also the subsurface atomic environment plays a crucial role in determining catalytic performance. While surface sites are directly exposed to reactants and often considered the primary active centers, subsurface atoms can modulate electronic structures, adsorption energies, and charge transfer pathways. This subsurface environment may therefore indirectly influence the binding of intermediates, the stability of active sites, and the overall catalytic activity. Modifications in the subsurface region (such as lattice strain, oxygen vacancies,^9,30–32^ or foreign atoms^7,33^ ) can alter the electronic density of surface atoms, thereby tuning the activity and selectivity of the catalyst. Understanding the interplay between surface and subsurface environments is thus essential for rational catalyst design and for elucidating the true origin of catalytic activity in complex oxide systems. This section will specifically address the role of the subsurface environment, highlighting how structural and electronic features below the surface contribute to catalytic activity and influence reaction pathways.

### Formation and Characterization of the Photocatalyst NiMoO_4_

NiMoO_4_ was synthesized via a simple and rapid microwave-assisted method. In this synthesis, prior to thermal treatment, the material exhibits a diffraction pattern like that of the material treated at 300 °C in a conventional muffle furnace (see Figure 2A). This pattern corresponds to NiMoO_4_·xH_2_O with a monoclinic structure, generally referred to as the α-phase of the material,^34^ with crystallographic data ICSD No. 247435. The main diffraction peaks observed at approximately 27.1° and 29.6° were indexed to the (021) and (122) crystallographic planes of NiMoO_4_·xH_2_O, respectively, indicating the formation of the hydrated phase. No secondary diffraction peaks associated with impurity phases such as NiO or MoO_3_ were detected, suggesting good phase purity of the synthesized material. In addition, the relatively broad diffraction peaks indicate the low crystallinity and nanostructured nature of the hydrated sample, which is consistent with the hydrothermal synthesis of hydrated molybdate materials.

**Figure 2:**
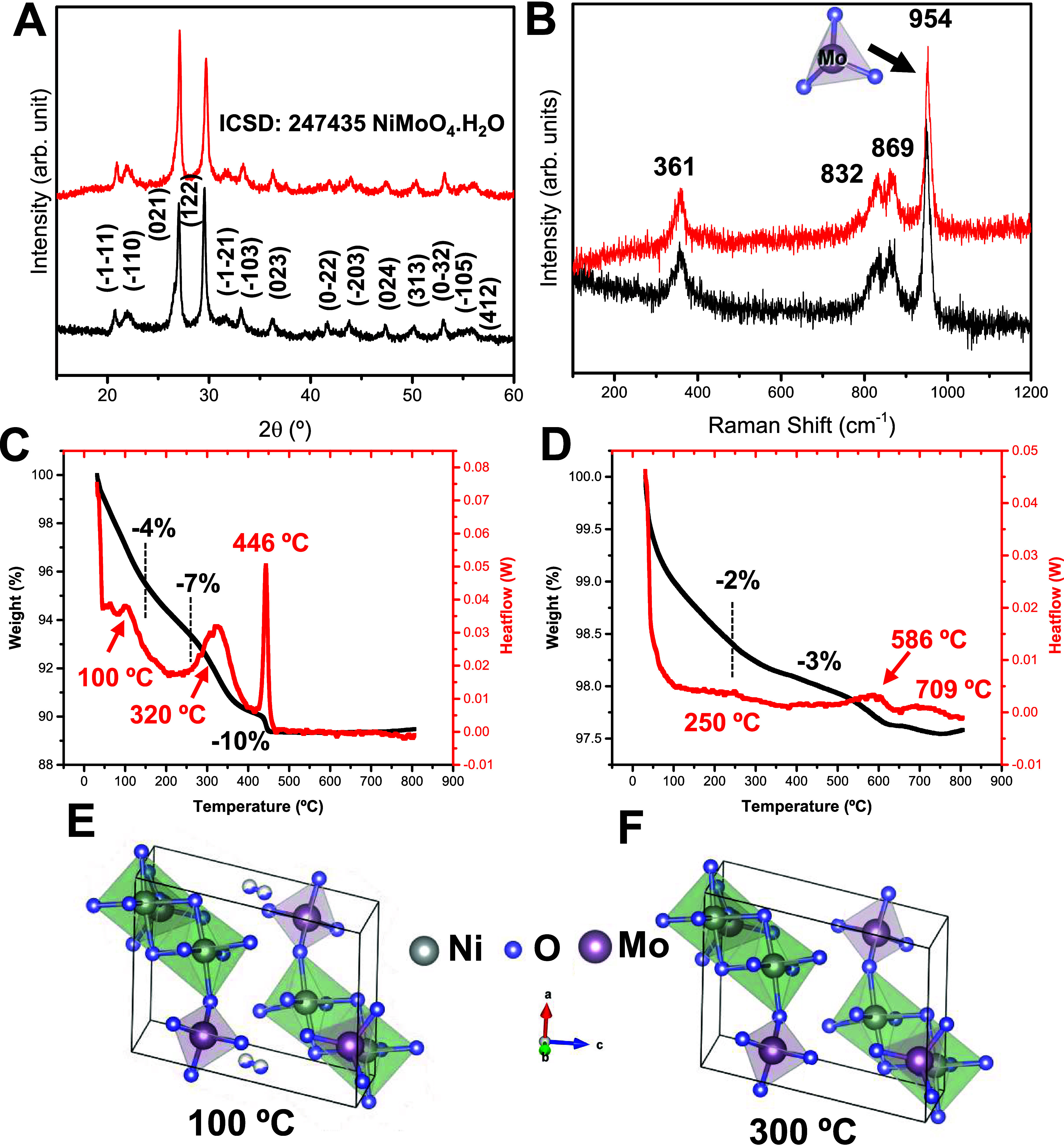
(A, B) XRD patterns and Raman of NiMoO_4_-100 °C (black line) and NiMoO_4_-300 °C (red line) catalysts. TG and DSC plots of (C) NiMoO_4_-100 °C and (D) NiMoO_4_-300 °C catalysts. Unit cell of the NiMoO_4_.xH_2_O structure (E, F) NiMoO_4_ structure.

Raman spectroscopy, in Figure 2B, further confirms this structural similarity, showing characteristic peaks at 954 cm^–1^ attributed to [MoO_4_] clusters in the NiMoO_4_ structure, along with asymmetric stretches at 869 cm^–1^ and 832 cm^–1^ associated with O–Mo–O bond vibrations within these clusters, and a peak at 361 cm^–1^ corresponding to cluster deformations.^35,36^ Considering that the main difference between the two materials is the calcination applied to the second sample, TG/DSC analyses were conducted to assess the behavior of the hydrated structure. The FTIR spectrum of NiMoO_4_-300 °C (Supporting Information, Figure S1) exhibits the characteristic vibrational bands of the molybdate structure in the 1000–500 cm^–1^ region, assigned to Mo = O, Mo–O–Mo, and Ni–O–Mo vibrations. Weak and broad bands observed near ∼3420 and ∼1630 cm^–1^, associated with O–H stretching and H–O–H bending vibrations, respectively, suggest the presence of structurally confined and/or adsorbed water with low relative intensity. As shown in Figure 2C,D, the untreated material (NiMoO_4_-100 °C) exhibits a progressive mass loss throughout the heating process. An initial weight reduction of approximately 4% is observed below 150 °C, reaching nearly 7% around 270 °C. Upon further heating to approximately 350 °C, the total mass loss reaches about 10%, indicating successive thermal events during the decomposition process. In contrast, the material treated at 300 °C (NiMoO_4_-300 °C) shows only two stages: about 2% at 220 °C and 1% at 400 °C, totaling only 3% mass loss. These results indicate that NiMoO_4_-100 °C contains structural water released during the first mass-loss stage, which is absent in NiMoO_4_-300 °C. Although both materials are hydrated, the amount of water in the structure of NiMoO_4_-100 °C is significantly higher than in the calcined material.^37^ More pronounced events at higher temperatures (≈400–700 °C) indicate structural transformations, accompanied by peaks in the heat flow curves, suggesting endothermic processes related to different crystalline reorganization.^37,38^ Differences between the samples, such as shifts of these events to higher temperatures, point to variations in thermal stability, likely associated with structural differences. Figure 2E,F presents the unit cells of NiMoO_4_·xH_2_O and anhydrous NiMoO_4_, respectively. Both structures adopt a monoclinic unit cell composed of [NiO_6_] octahedra and [MoO_4_] tetrahedra.

The presence of water molecules in the crystalline structure can induce local disorder, favoring the formation of structural and electronic defects such as vacancies, bond distortions, and intermediate states within the bandgap.^39–42^ Such defects are often investigated by PL, since radiative recombination processes are highly sensitive to the electronic levels enabled by these imperfections. To evaluate these defect-mediated electronic transitions, the materials were excited with a 355 nm laser, stimulating recombination processes related to intragap states. Figure 3(A,B) shows the PL spectra of NiMoO_4_-100 °C and NiMoO_4_-300 °C, respectively. The spectra exhibit broad bands, typical of semiconductors containing multiple emitting centers. Deconvolution of these bands makes it possible to separate the contributions associated with different intragap states, allowing the energy corresponding to each radiative recombination process to be inferred. This broad emission results from the superposition of electronic transitions involving blue shift (∼2.7 eV) shallow defects (located near the valence or conduction band) and red shift (∼2.1 eV) deep defects, which lie farther from the band edges. In comparison, the NiMoO_4_-100 °C material shows higher intensity attributed to deep defects. This suggests that structural water present in this material promotes a higher density of defect states, which aligns with observations in hydrated oxides and materials with greater octahedral distortion (frequently discussed in studies of transition metal oxides containing coordination water).^43,44^ From a functional standpoint, such defects can act as charge-trapping centers and also serve as active sites that facilitate the adsorption and activation of N_2_ molecules on the catalyst surface under illumination. Thus, the higher defect density observed in NiMoO_4_-100 °C may contribute to improved photoreduction processes, reinforcing the role of water-induced disorder in modulating the semiconductor’s photophysical properties.^45^

**Figure 3:**
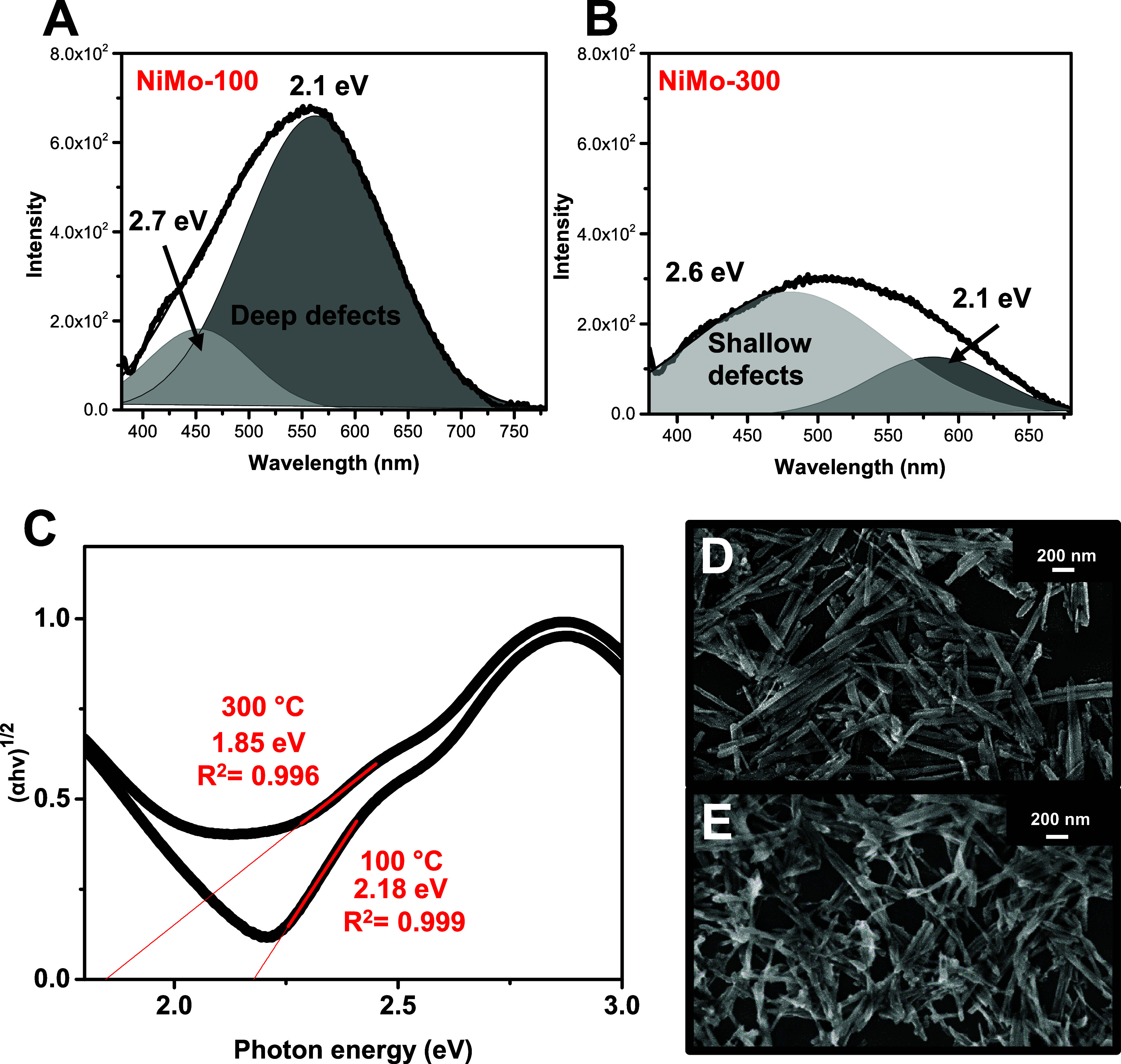
(A, B) PL emission spectra, (C) band gap analysis via DRS UV–vis, and SEM characterization of (D) NiMoO_4_-100 °C and (E) NiMoO_4_-300 °C catalysts.

DRS measurements used to estimate the band gap reveal noticeable differences between the structurally modified materials. In Figure 3C, the samples NiMoO_4_-100 °C and NiMoO_4_-300 °C exhibit band gap values of 2.18 and 1.85 eV, respectively. This variation indicates that, despite their similar rod-like morphology observed in Figure 3(D,E), significant changes occur at the electronic structure level. Such morphology is characteristic of monoclinic NiMoO_4_ and suggests that morphological effects alone cannot account for the observed band gap differences. The reduction in band gap for NiMoO_4_-300 °C may be associated with changes in crystallinity induced by thermal treatment and reduced deep defect density, which can improve charge transport but simultaneously decrease the number of catalytically active defect sites involved in N_2_ reduction. Although both materials share similar morphological features, the band gap reflects not only surface facets but also bulk electronic structure and defect-related states.^46^ The DRS and PL results further indicate differences in the charge carrier recombination behavior of the samples, with NiMoO_4_-300 °C showing more favorable charge carrier dynamics. However, despite this improved charge separation behavior, the superior photocatalytic nitrogen reduction performance observed for NiMoO_4_-100 °C suggests that additional factors, particularly defect-related surface states and adsorption properties, play a crucial role in the catalytic process. In this context, the higher density of deep-level defect states in NiMoO_4_-100 °C may favor N_2_ adsorption and activation, contributing more significantly to the photocatalytic activity than charge carrier separation alone.

### Evaluation of Nitrogen Reduction: Influence of Reaction Parameters on NiMoO_4_ Catalysts with High and Low Structural Water Content

To evaluate the photocatalytic performance of NiMoO_4_-100 °C and NiMoO_4_-300 °C in N_2_ reduction, control experiments were conducted under both dark and illuminated conditions, under Ar atmosphere, and in the absence of a catalyst. As shown in Figure 4, the control experiment resulted in NH_3_ values classified as N/D (not detected), demonstrating that no significant ammonia formation occurred under these conditions. The NiMoO_4_-100 °C catalyst, as well as experiments conducted under both dark and illuminated conditions in the absence of a catalyst, resulted in NH3 values classified as N/D (not detected), demonstrating that no significant ammonia formation occurred under these control conditions. The NH_3_ concentration profiles indicate that NiMoO_4_-100 °C produces 4.2 μmol L^–1^ of NH_3_ in the dark and 13 μmol L^–1^ under illumination. This ∼3-fold increase upon exposure to blue LED light (385 nm) demonstrates the photocatalytic capability of this material.

**Figure 4:**
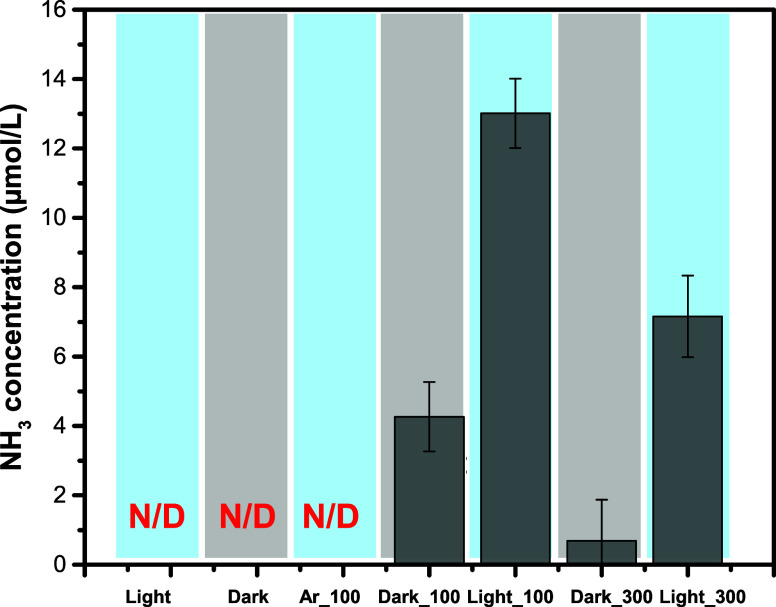
Ammonia production in water via photocatalytic argon and nitrogen reduction under blue LED light (LED λ = 385 nm; triplicate data sets) and dark in NiMoO_4_-100 °C and NiMoO_4_-300 °C catalysts. Reaction conditions: 100 mL of solvent (H_2_O), 50 mg of catalyst, and a reaction time of 2 h (light = blue color or dark = gray color).

In contrast, NiMoO_4_-300 °C exhibits lower activity, producing 0.7 μmol L^–1^ of NH_3_ in the dark and 7.1 μmol L^–1^ under light irradiation. Although this corresponds to an approximately 10-fold increase, the absolute NH_3_ production under illumination remains lower than that of NiMoO_4_-100 °C. These results suggest that NiMoO_4_-100 °C, which likely contains a higher structural water content and a greater density of deep defect states, may promote more efficient and selective N_2_ reduction. Although NiMoO_4_-300 °C exhibited a narrower band gap and enhanced visible-light absorption, this characteristic alone did not lead to superior photocatalytic ammonia production. The results suggest that the photocatalytic performance is more strongly influenced by the nature and distribution of defect-related active sites than solely by band gap narrowing. In this regard, NiMoO_4_-100 °C, despite presenting a wider band gap, likely contains a higher concentration of deep defect states that can facilitate N_2_ adsorption and activation on the catalyst surface, contributing to its improved photocatalytic activity.

It is important to note that NH_3_ formation under dark conditions is not necessarily expected to be completely negligible in nitrogen reduction systems, since adsorption and surface-mediated catalytic processes may still occur in the absence of light irradiation. In particular, the NiMoO_4_-100 °C sample exhibited a higher adsorption capacity compared to NiMoO_4_-300 °C, which may favor the interaction and activation of nitrogen-containing species at the catalyst surface, contributing to the NH_3_ signal observed under dark conditions. Nevertheless, control experiments performed in the absence of a catalyst, both under dark conditions and light irradiation, resulted in negligible NH_3_ formation, confirming that ammonia production is directly associated with the presence of the catalytic material.

The results presented in Figure 4 were obtained in the absence of a hole scavenger, using only an aqueous solution at pH 7. To further investigate the role of photogenerated holes in charge separation under LED irradiation, methanol was introduced as a quencher. Methanol acts as a hole scavenger, reacting with the hole-rich regions of the semiconductor surface and thereby enhancing the availability and lifetime of photogenerated electrons for reduction reactions. Figure 5A shows the NH_3_ production for both photocatalysts under LED irradiation as a function of methanol concentration (0, 0.01, 0.1, and 0.5 mol L^–1^). A clear trend is observed for both materials: the addition of methanol improves photocatalytic activity up to an optimal concentration of 0.1 mol L^–1^ (27.5 μmol L^–1^ NH_3_). Beyond this point, higher methanol concentrations may contribute to the observed decrease in photocatalytic performance, possibly due to oxidation products such as HCHO, HCOOH, or CO_2,_ which may interfere with interfacial adsorption processes or partial saturation of active sites on the catalyst surface.^47,48^ At excessive concentrations, as observed for Tahir et al., methanol can compete with reactants for active sites, alter interfacial adsorption dynamics, and even promote side reactions, ultimately reducing selectivity toward N_2_ reduction. Similar behavior was reported by Tahir et al., who observed improved H_2_ production for cobalt–carbon nitride systems using 5% methanol, while higher alcohol concentrations (10% methanol) led to a decrease in activity.^47^ Across all methanol concentrations, NiMoO_4_-100 °C consistently exhibits higher NH_3_ production than NiMoO_4_-300 °C, indicating that its surface provides a greater number of active sites for N_2_ reduction. This enhanced performance can be associated with its higher structural water content and, importantly, a greater density of deep defect states. Deep defects play a crucial role in photocatalysis because they can act as charge carrier traps, particularly for electrons. These trap states can prolong the lifetime of photogenerated electrons by preventing rapid recombination with holes. In the context of N_2_ reduction, this is especially beneficial, as the reaction requires multiple electrons and is kinetically demanding. Therefore, the presence of deep defect-related states in NiMoO_4_-100 °C may contribute to electron accumulation at the surface and influence the N_2_ adsorption/activation process, which could be associated with the enhanced photocatalytic nitrogen reduction performance observed for this material. However, unlike shallow defects, deep defects may also influence adsorption properties and local electronic structure, potentially stabilizing reaction intermediates. This combination of improved charge separation and favorable surface chemistry helps explain the superior photocatalytic performance of NiMoO_4_-100 °C compared to NiMoO_4_-300 °C. The proposed mechanism involves photoexcitation of NiMoO_4_·xH_2_O under blue LED light, generating electron–hole pairs, see Figure 5B; effective charge separation; (1) oxidation of methanol by photogenerated holes, producing protons and intermediates; and (2) multielectron reduction of N_2_ to NH_3_ by photogenerated electrons.

**Figure 5:**
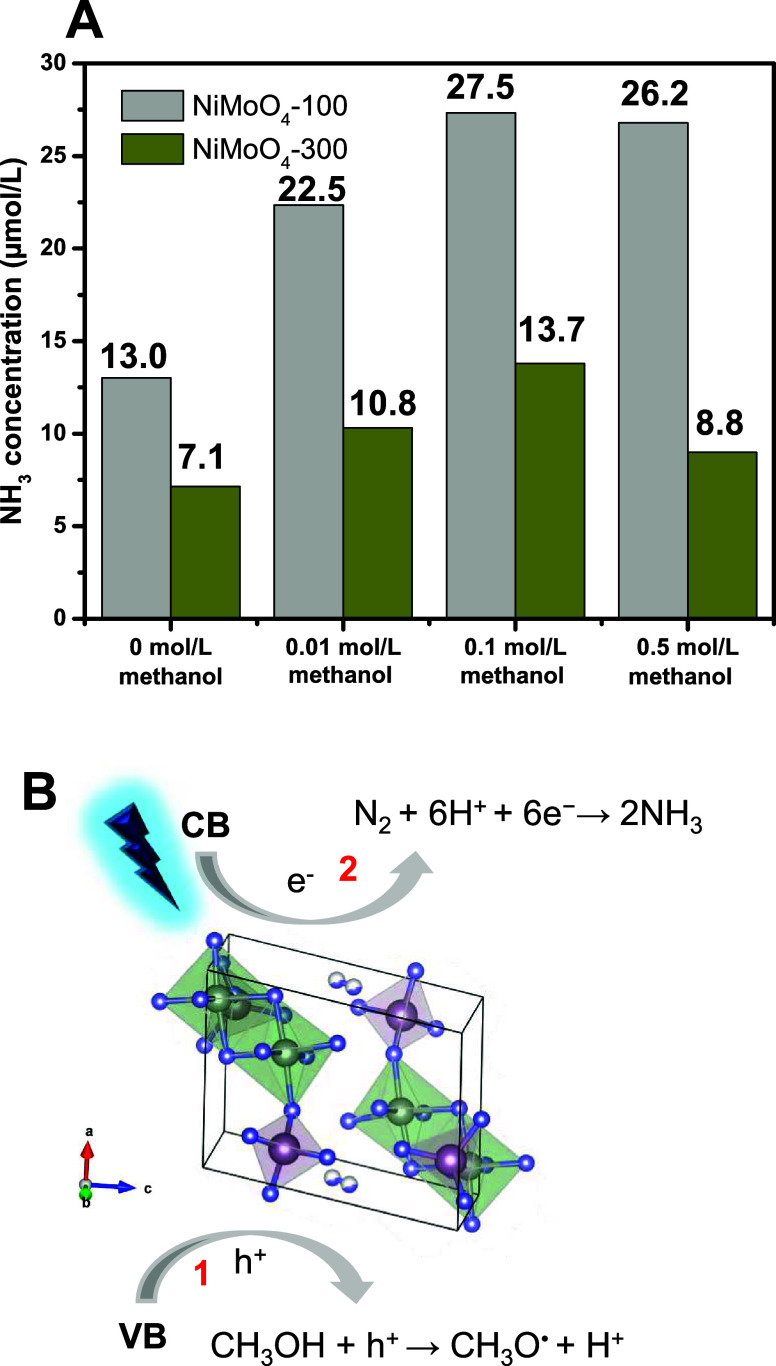
(A) Ammonia production via photocatalytic nitrogen reduction under blue LED light (LED 385 nm) in NiMoO_4_-100 °C (gray) and NiMoO_4_-300 °C (green) catalysts with different amounts of methanol as a quencher. Reaction conditions: 100 mL of solvent (H_2_O), 50 mg of catalyst, and a reaction time of 2 h (light). (B) Photoinduced charge separation-assisted pathway for N_2_ reduction and methanol oxidation mediated by NiMoO_4._.

### Surface Environment Contributions to Catalytic Activity

The catalytic surface, together with its immediate chemical environment, represents the primary interface at which reactions occur. Beyond the intrinsic properties of surface atoms, the surrounding environment, including adsorbed species, solvent interactions, solvent composition, and local pH, plays a decisive role in shaping catalytic behavior. These factors govern the adsorption and desorption of reactants, stabilize key intermediates, and can either promote or hinder charge transfer processes. This section will specifically address how the surface environment contributes to catalytic activity, emphasizing the interplay between the surface atomic structure and the dynamic chemical conditions at the interface.

### Reduction of Nitrogen: Effect of the Polymer of Intrinsic Microporosity

PIM-EA-TB was evaluated as a surface environment modifier to assess its contribution to catalysis in combination with NiMoO_4_.xH_2_O. In this approach, 1 mg of PIM-EA-TB was applied as a coating on the surface of 50 mg NiMoO_4_-100 °C, which previously demonstrated superior NH_3_ production. The experiments were conducted in aqueous media under near-neutral pH conditions. Under these conditions, significant protonation of PIM-EA-TB is not expected. Figure 6 presents a comparison between NiMoO_4_-100 °C and PIM-EA-TB-coated NiMoO_4_-100 °C. The results show a substantial increase in NH_3_ production, from 12 μmol L^–1^ for the unmodified catalyst to 32.8 μmol L^–1^ for the PIM-coated system, corresponding to an approximately 2.7-fold enhancement. This improvement the positive role of PIM-EA-TB in promoting the efficiency of N_2_ reduction. Although local proton generation may occur during photocatalysis (in photoanode regions), these effects are transient and insufficient to induce stable protonation of the polymer. The enhanced catalytic performance could be attributed to interfacial effects, including proton capture for photoanodic processes and the reuse of these protons locally in the photocathodic ammonia production. The possible contribution of nitrogen from the PIM-EA-TB directly to the origin of the produced ammonia was confirmed by isotope studies (*vide infra*).

**Figure 6:**
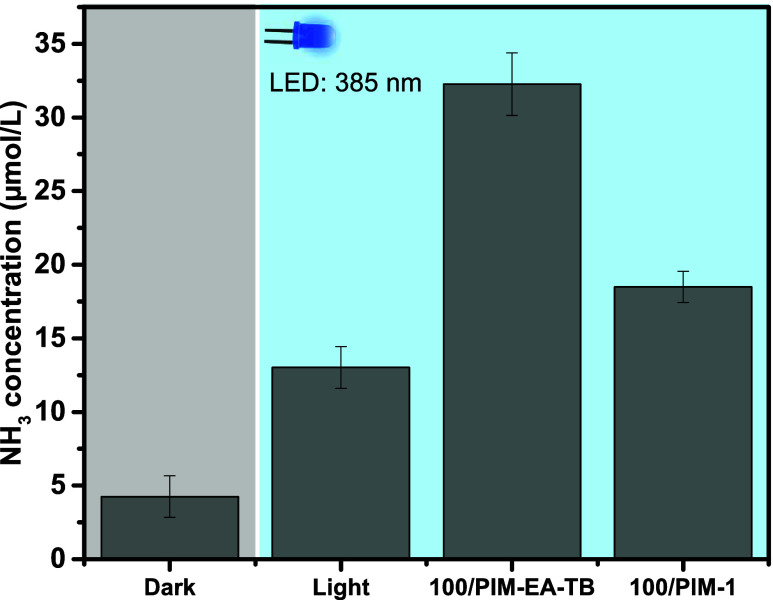
Ammonia production via photocatalytic nitrogen reduction in water under blue LED light (LED λ = 385 nm; triplicate measurements) and dark in NiMoO_4_-100 °C pure and NiMoO_4_-100 °C coating with 1 mg PIM-EA-TB and PIM-1 catalysts. Reaction conditions: 100 mL of solvent (H_2_O), 50 mg of catalyst, and a reaction time of 2 h (light or dark).

Electrochemical studies on MoS_2_ coated with PIM-1, as reported by Almeida et al.,^24^ demonstrated that NH_3_ production efficiency was approximately two times higher compared to that on bare MoS_2_. The authors attributed this enhancement primarily to the ability of PIM-1 to confine N_2_ molecules within its microporous structure, increasing the local concentration of reactants near active sites. A similar trend is observed in the present work, where the incorporation of PIM-EA-TB leads to a significant improvement in NH_3_ production. However, an important distinction must be emphasized: unlike the electrochemical system reported for PIM-1, the experiments in this study were conducted under photocatalytic conditions, in the absence of an applied external potential. Therefore, both photoanodic and photocathodic processes occur in close vicinity. Interestingly, the incorporation of PIM-1 and NiMoO_4_-100 °C resulted in lower photocatalytic activity compared to PIM-EA-TB, suggesting that not all PIM structures contribute equally to ammonia production.

In this context, the role of PIM-EA-TB extends beyond gas confinement. Due to its more rigid structure and the presence of nitrogen-containing functional groups (p*K*_A_ ∼ 4), PIM-EA-TB can facilitate interfacial charge transfer and modulate the local reaction environment. These features are particularly relevant in photocatalysis, where charge separation and transport are critical limiting factors.

The results presented in Figure 7A,B show the production of NH_3_ under different concentrations of PIM-EA-TB on NiMoO_4_-100 °C (0, 1, 2, and 5 mg PIM-EA-TB with 50 mg NiMoO_4_-100 °C). A clear trend is observed: excessive amounts of PIM-EA-TB lead to a decrease in catalytic performance. A concentration of 5 mg results in a reduced NH_3_ production of 6.9 μmol L^–1^, which is even lower than that of pure NiMoO_4_, indicating that excessive polymer coating induces a detrimental effect. With an intermediate concentration of 2 mg, NH_3_ production drops 18.8 μmol L^–1^, showing an improvement compared to the pure material. However, optimal performance is achieved with 1 mg of PIM-EA-TB, which provides the highest NH_3_ production. These results suggest that there is an ideal balance between surface modification and accessibility of active sites. To better understand the effect of increasing the PIM-EA-TB loading, SEM analyses were performed. Figure 7C–K show the morphology of the materials coated with 1 mg (Figure 7C–E), 2 mg (Figure 7F–H), and 5 mg (Figure 7I–K) at different magnifications (25×, 50×, and 100×). The SEM images suggest that the incorporation of PIM-EA-TB leads to the progressive aggregation of the NiMoO_4_-100 °C particles compared to SEM in Figure 3D. With a lower loading (1 mg), the material appears relatively better dispersed. In contrast, increasing the polymer content promotes the formation of larger and more compact aggregates. At 5 mg, the material appears significantly agglomerated, indicating that excessive polymer coating may hinder the dispersion of the active particles. Based on the morphological observations, the deposited material appears to partially cover the catalyst surface rather than forming a complete and uniform monolayer. Although SEM analysis primarily provides morphological information, these observations suggest that excessive PIM-EA-TB coating may reduce the accessibility of active sites and limit mass transfer, which could explain the decrease in NH_3_ production at higher concentrations. Therefore, better performance with lower PIM-EA-TB content is likely associated with a more favorable balance between surface modification and the availability of catalytic sites, and not just morphological changes. A control experiment using only PIM-EA-TB (1 mg) under the same conditions showed no detectable ammonia formation by LC–MS, indicating that no reduction reaction occurred in the absence of the catalyst. This result confirms that the catalyst plays a key role in electron generation required for N_2_ reduction.

**Figure 7:**
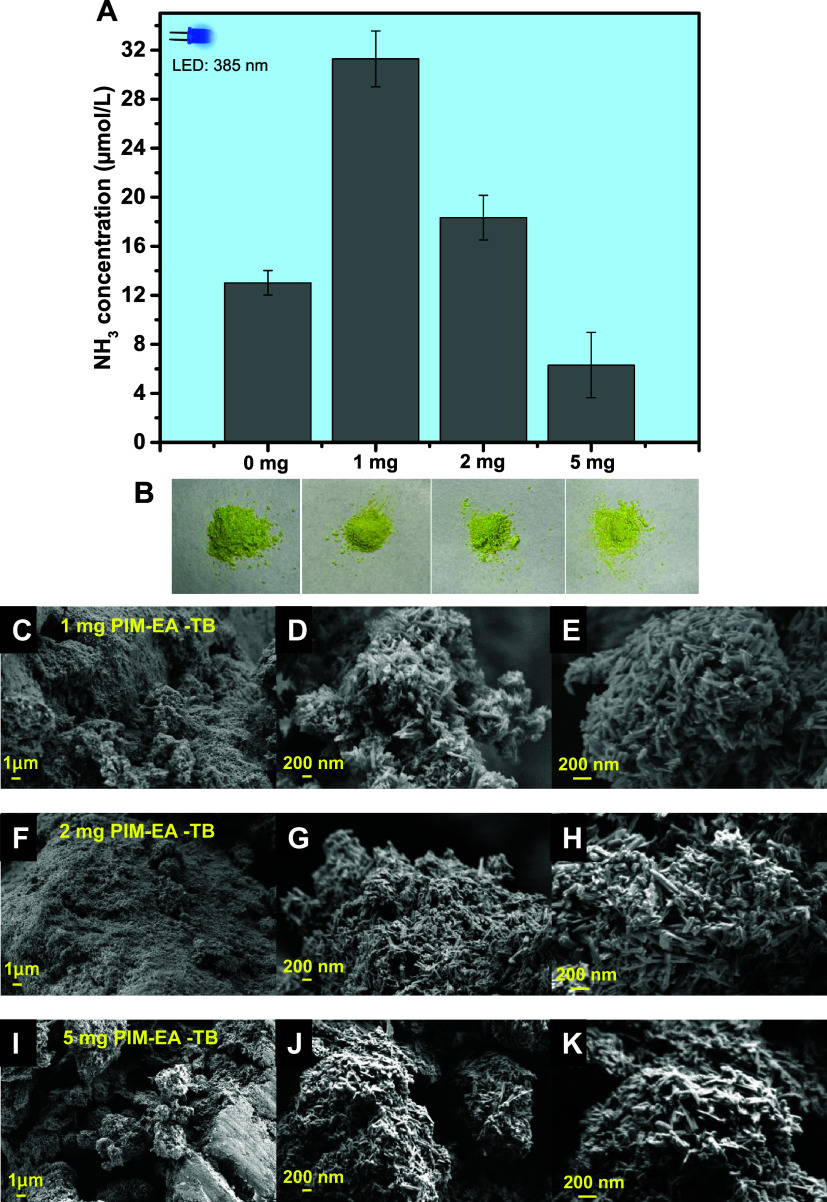
(A) Ammonia production via photocatalytic nitrogen reduction in water under blue LED light (LED 385 nm; three repeats) and pure (0 mg) and NiMoO_4_-100 °C coating with 1 mg, 2 mg and 5 mg of the PIM-EA-TB polymer. Reaction conditions: 100 mL of solvent (H_2_O), 50 mg of catalyst, and a reaction time of 2 h (light). (B) Below each catalyst amount, photographs of the resulting NiMoO_4_-100 °C and PIM-EA-TB powders are shown. SEM images of NiMoO_4_/PIM-EA-TB composites. (C–E) 1 mg of PIM-EA-TB/NiMoO_4_-100 °C, (F–H) 2 mg of PIM-EA-TB/NiMoO_4_-100 °C, and (I–K) 5 mg of PIM-EA-TB/NiMoO_4_-100 °C.

EDS analysis was performed for the NiMoO_4_-100 °C sample containing 1 mg of PIM-EA-TB (Supporting Information, Figure S2). The EDS confirmed the presence of Ni (20.2%), Mo (26.9%), and O (26.3%), consistent with the nickel molybdate phase, together with C (26.2%) and N (0.5%) signals attributed to the PIM-EA-TB polymer. The comparatively lower intensity of the C and N peaks relative to the inorganic elements suggests that the polymer is present in a relatively small amount and is mainly distributed as a thin surface coating on the NiMoO_4_ particles rather than forming a dominant bulk phase. These results support the proposed role of PIM-EA-TB as an inert interfacial layer in the material.

The use of methanol as a hole scavenger further enhanced the N_2_ reduction performance of the NiMoO_4_-based system and was therefore evaluated in combination with the polymer. As shown in Figure 8A, a consistent trend of increased NH_3_ production is observed. Specifically, the NH_3_ concentration rises from 13.0 μmol L^–1^ for pristine NiMoO_4_-100 °C to 32.3 μmol L^–1^ for NiMoO_4_-100 °C coated with PIM-EA-TB, and further to 42.2 μmol L^–1^ upon the addition of methanol, corresponding to an approximately 1.3-fold enhancement relative to the polymer-coated system. This improvement can be attributed to the role of methanol in scavenging photogenerated holes, thereby suppressing electron–hole recombination and increasing the availability of electrons for the reduction of N_2_. Additionally, the consumption of holes may lead to the generation of protons and induce local pH variations at the NiMoO_4_/PIM-EA-TB interface. Such interfacial changes can favor proton-coupled electron transfer steps, which are essential for efficient N_2_ reduction to NH_3_. Therefore, the combined effect of improved charge separation and modulation of the local reaction environment/pH contributes to the enhanced photocatalytic performance observed in the presence of methanol.

**Figure 8:**
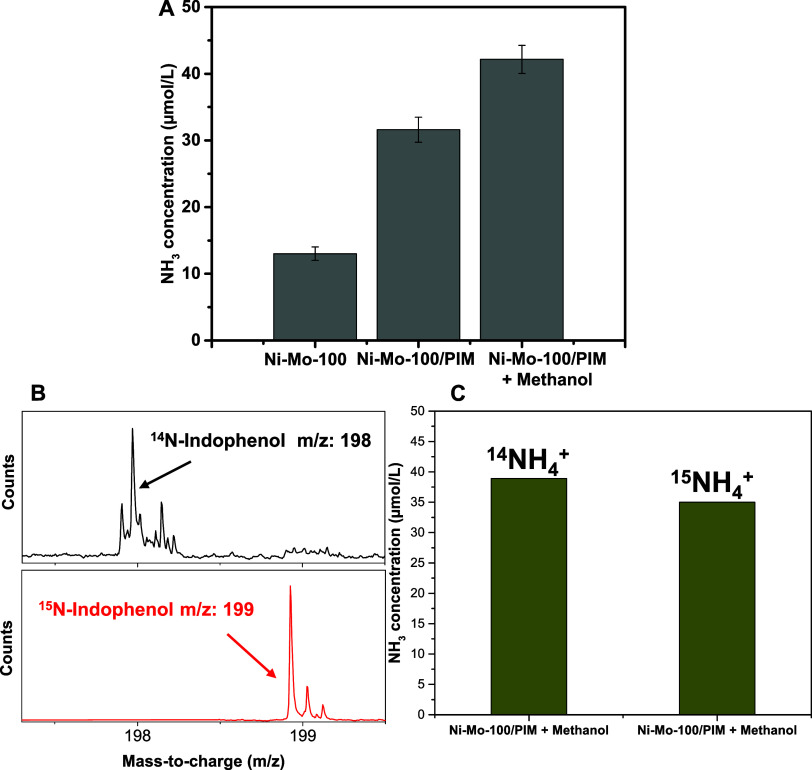
(A) Ammonia production via photocatalytic nitrogen reduction under blue LED light (LED λ = 385 nm) in NiMoO_4_-100 °C catalysts with PIM-EA-TB and 0.1 mol L^–1^ of methanol. Reaction conditions: (1) NiMoO_4_-100 °C and (2) NiMoO_4_-100/PIM: 100 mL H_2_O, 50 mg of catalyst, and a reaction time of 2 h (light); (3) NiMoO_4_-100/PIM + Methanol: 96 mL H_2_O + 0.1 mol L^–1^ of methanol, 50 mg of catalyst, and a reaction time of 2 h (light). (B, C) GC–MS spectra of ammonia produced under ^14^N_2_ and ^15^N_2_ atmospheres.

To verify the consistency and origin of ammonia production in this system, isotopic labeling experiments using ^15^N_2_ were performed. The LC–MS results, presented in Figure 8B,C, compare the products obtained using ^14^N_2_ and ^15^N_2_. The data clearly indicates that, in the absence of ^14^N_2_, the detected NH_3_ originates exclusively from the reduction of the supplied ^15^N_2_ during the photocatalytic reaction. These results confirm that the observed ammonia production is directly associated with N_2_ reduction rather than contamination. Furthermore, they demonstrate that the PIM-EA-TB polymer, despite containing nitrogen-based functional groups, does not undergo degradation or contribute to nitrogen contamination under the reaction conditions. Similar observations have been reported for PIM-1 ^24^ under comparable conditions (pH ≈ 7, phosphate buffer), where ^15^N_2_ labeling experiments also confirmed that the produced NH_3_ originates from N_2_ reduction. These findings suggest that PIM-based materials, in general, are stable under such conditions and can be reliably employed as coatings in ammonia production systems without introducing nitrogen-related artifacts.

These results confirm that the observed ammonia production is directly associated with N_2_ reduction, rather than contamination. Furthermore, the results demonstrate that the PIM-EA-TB polymer, despite containing nitrogen-based functional groups, does not undergo degradation or contributes to nitrogen contamination in the system.

Based on the results obtained for photocatalytic nitrogen fixation to ammonia, Table 1 presents a comparison with previously reported materials for N_2_ photoreduction. It is important to note that the photocatalytic ammonia production values reported in Table 1 were obtained under different irradiation conditions, including blue LED, UV–vis, and simulated solar-light sources. Since the spectral distribution and photon flux of these light sources may differ significantly, a direct comparison of the photocatalytic activities should be made with caution. Therefore, the comparison presented here is intended only as a qualitative reference to the previously reported photocatalytic systems.

**Table 1:** Photocatalytic Performance of Different Catalysts for N_2_ Reduction in Aqueous Medium

photocatalyst	light source	reactants (mL min^–1^)	concentration of photocatalyst	ammonia production rate^a^	quencher	ammonia detection methods	refs
In–Bi_2_MoO_6_	simulated sunlight	N_2_/H_2_O	100 mg/100 mL	53.4 μmol g_cat_^–1^ h^–1^	N.A.	Nessler’s reagent	^52^
KNbO_3_@TMU-5	UV–vis	N_2_/H_2_O	10 mg/10 mL	39.9 μmol L^–1^g_cat_^–1^ h^–1^	ethanol	indophenol indicator	^53^
KNbO_3_	UV–vis	N_2_/H_2_O	10 mg/10 mL	20.5 μmol L^–1^ g_cat_^–1^ h^–1^	ethanol	indophenol indicator	^53^
TiO_2_@LSM	simulated sunlight	N_2_/H_2_O	10 mg^b^	5.41 mg L^–1^ g_cat_^–1^ h^–1^	N.A.	salicylate method	^51^
AuNP/n-Bi_2_O_3–*x*_	simulated sunlight	N_2_/H_2_O	10 mL^c^	432.5 μmol g_cat_^–1^ h^–1^	0.01% SDS	indophenol indicator	^50^
W-doped Sb_2_OS_2_	simulated sunlight	N_2_/H_2_O	50 mg/50 mL	408.1 μmol g_cat_^–1^ h^–1^	N.A.	Nessler’s reagent, indophenol blue method, and ion chromatography	^49^
TiO_2_@PTFE	simulated sunlight	N_2_/H_2_O	10 mg/80 mL	133.6 μmol g_cat_^–1^ h^–1^	N.A.	indophenol indicator	^22^
NiMoO_4_.xH_2_O	blue LED	N_2_/H_2_O	50 mg/100 mL	130.2 μmol g_cat_^–1^ h^–1^	no scavenger	indophenol indicator	this work
NiMoO_4_.xH_2_O-PIM-EA-TB	blue LED	N_2_/H_2_O	50 mg/100 mL	322.6 μmol g_cat_^–1^ h^–1^	no scavenger	indophenol indicator	this work
NiMoO_4_.xH_2_O-PIM-EA-TB	blue LED	N_2_/H_2_O	50 mg/100 mL	423.7 μmol g_cat_^–1^ h^–1^	0.1 mol/L methanol	indophenol indicator	this work

a Units are reported as originally presented in the publication, without conversion or standardization.

b No information about the volume used.

c No precise information on the mass used.

Wu et al.^49^ investigated oxygen vacancy defects in W-doped Sb_2_OS_2_ under simulated solar irradiation, achieving an NH_3_ production rate of 408.1 μmol g^–1^ h^–1^. Similarly, Prabagar, Reddy and Lim^50^ reported Au/Bi_2_O_3‑x_ nanoparticles for efficient N_2_-to-NH_3_ conversion, reaching 432.5 μmol g_cat_^–1^ h^–1^. In another study, Ma et al.^51^ reported TiO_2_@layered silicate (magadiite) nanosheets, achieving 5.41 mg L^–1^ g_cat_^–1^ h^–1^ under combined piezo-photocatalysis conditions. In a related approach, Shi et al.^22^ reported a synergistic TiO_2_@PTFE system, achieving an enhancement of up to 10.9 times compared to pristine TiO_2_. This result further supports the concept that coupling photocatalysis with interfacial strategies can significantly boost catalytic performance. Compared to these systems, the incorporation of PIM-EA-TB significantly enhances the performance of NiMoO_4_·xH_2_O, improving its competitiveness among state-of-the-art materials for photocatalytic N_2_ reduction.

## Conclusion

NiMoO_4_ has been synthesized and is shown to produce ammonia from N_2_ in water under blue LED illumination. Changes in the crystal structure and defect population due to the loss of water during annealing clearly detrimentally impact the catalyst performance. However, the presence of methanol, acting as a hole quencher, is shown to significantly improve ammonia production. Deposition of microporous polyamine (PIM-EA-TB) on the surface of the NiMoO_4_ particles further increases the yield of the photocatalytic reaction. With the degradation of the polymer to ammonia being ruled out by ^15^N_2_ isotope experiments, the proposed role of PIM-EA-TB is to capture protons from photoanodic processes, allowing their reuse in photocathodic ammonia production. This mechanism is related to a previously reported PIM-EA-TB effect on the catalytic H_2_O_2_ formation with embedded palladium catalyst particles.^28^ Further study is necessary to systematically elucidate similar surface modification effects on photocatalytic ammonia production.

## Supplementary Material



## Notes

FTIR spectrum of NiMoO_4_-100 °C and NiMoO_4_-300 °C samples; EDS analysis of the NiMoO_4_-100 °C/PIM-EA-TB (PDF)

## ⊥

L.K.R: Institute of Chemistry, University of São Paulo, São Paulo 05508-000, Brazil

## Abbreviations

PIM: polymers of intrinsic microporosity
PIM-1: polymer of intrinsic microporosity-1
PIM-EA-TB: polymer of intrinsic microporosity based on ethanoanthracene and Tröger’s Base
LED: light emitting diode
PTFE: poly­(tetrafluoroethylene)
FFV: fractional free volume
ICSD: inorganic crystal structure database
CCD: charge-coupled device
PL: photoluminescence
TG/DSC: thermogravimetric analysis/differential scanning calorimetry
SEM: scanning electron microscopy
DRS: diffuse reflectance spectroscopy
LC-MS: liquid chromatography–mass spectrometry
