# Supplementary material for: Microporous Polyamine (PIM-EA-TB) Modified with Hydrated NiMoO4 Enhances the Photocatalytic Reduction of Nitrogen to Ammonia
Source: ACS Appl Eng Mater. 2026 Jun 5;4(6):3457–70. doi: 10.1021/acsaenm.6c00493 (PMC13316990; doi:10.1021/acsaenm.6c00493)
Supplement: Supplementary file 1 [file em6c00493_si_001.pdf]

Supporting Information

Microporous Polyamine (PIM-EA-TB) Modified with Hydrated $NiMoO_4$ Enhances the Photocatalytic Reduction of Nitrogen to Ammonia

Lara K. Ribeiro [1,2*], Ana Beatriz Cardile [1,2], Laura O. Libero [1], Mariolino Carta [3], Neil B. McKeown [4], Lucia H. Mascaro [1*], Frank Marken [2]

[1] Department of Chemistry, Federal University of São Carlos São Carlos, SP, 13565-905, Brazil

[2] Department of Chemistry, University of Bath Claverton Down, Bath BA2 7AY, UK

[3] Instituto de Síntesis Química y Catálisis Homogénea, CSIC-Universidad de Zaragoza, C/Pedro Cerbuna 12, Facultad de Ciencias, Zaragoza 50009, Spain

[4] EaStCHEM, School of Chemistry, University of Edinburgh, Joseph Black Building, David, Brewster
Road, Edinburgh, EH9 3JF, Scotland, UK

Authors' email: larakribeiro@usp.com, lmascaro@ufscar.br

To be submitted to ACS Appl. Engineering Materials

Proofs to Lara Kelly Riberio (larakribeiro@gmail.com)

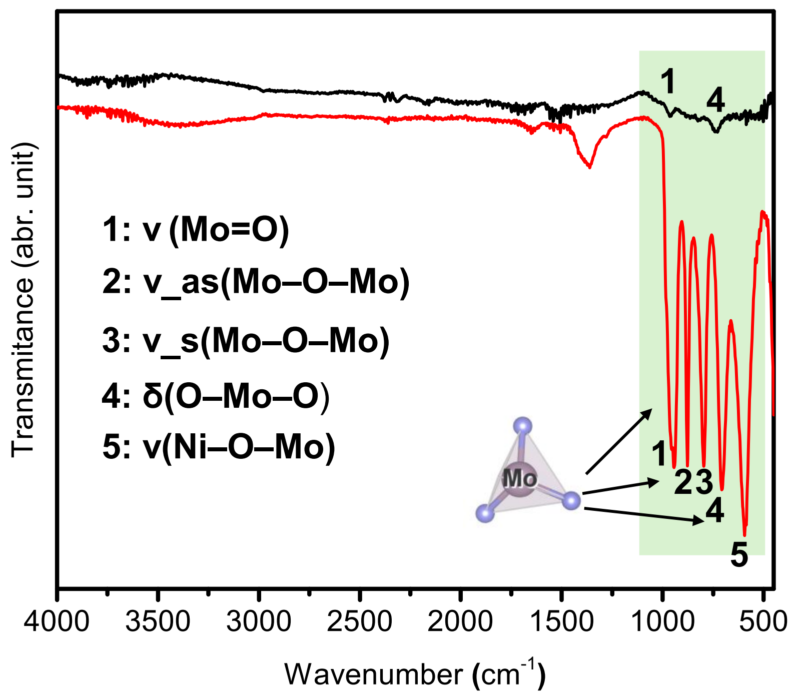

Figure S1. FTIR spectra of $NiMoO_4$ samples. The $NiMoO_4$-100 °C sample (black curve) and $NiMoO_4$-300 °C (red curve).

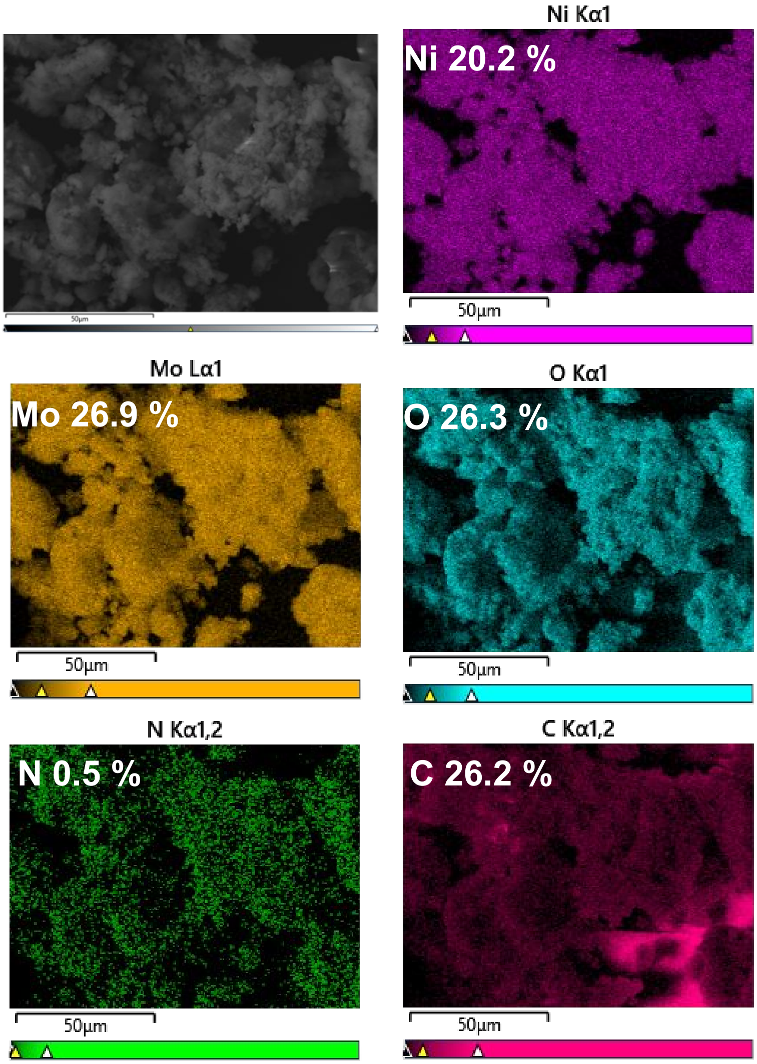

Figure S2. EDS spectrum of the NiMoO4-100 °C/PIM-EA-TB, showing the presence of Ni, Mo, O, N, and C.
